# Divergent Structural Responses to Pharmacological Interventions in Orbitofronto-Striato-Thalamic and Premotor Circuits in Obsessive-Compulsive Disorder

**DOI:** 10.1016/j.ebiom.2017.07.021

**Published:** 2017-07-26

**Authors:** Qiming Lv, Zhen Wang, Chencheng Zhang, Qing Fan, Qing Zhao, Kristina Zeljic, Bomin Sun, Zeping Xiao, Zheng Wang

**Affiliations:** a Institute of Neuroscience, Key Laboratory of Primate Neurobiology, CAS Center for Excellence in Brain Science and Intelligence Technology, Shanghai Institute for Biological Sciences, Chinese Academy of Sciences, Shanghai 200031, China; b University of Chinese Academy of Sciences, China; c Shanghai Mental Health Center, Shanghai Jiao Tong University School of Medicine, Shanghai 200030, China; d Shanghai Key Laboratory of Psychotic Disorders, China; e Department of Functional Neurosurgery, Shanghai Jiao Tong University School of Medicine, Shanghai 200025, China; f Shanghai Jiao Tong University School of Medicine, Shanghai 200025, China

**Keywords:** OCD, Drug-naïve, Pharmacotherapy, Voxel-based morphometry, Cross-validation

## Abstract

Prior efforts to dissect etiological and pharmacological modulations in brain morphology in obsessive-compulsive disorder (OCD) are often undermined by methodological and sampling constraints, yielding conflicting conclusions and no reliable neuromarkers. Here we evaluated alteration of regional gray matter volume including effect size (Cohen's *d* value) in 95 drug-naïve patients (age range: 18–55) compared to 95 healthy subjects (age: 18–63), then examined pharmacological effects in 65 medicated (age: 18–57) and 73 medication-free patients (age: 18–61). Robustness of statistical outcomes and effect sizes was rigorously tested with Monte Carlo cross-validation. Relative to controls, both drug-naïve and medication-free patients exhibited comparable volumetric increases mainly in the left thalamus (*d* = 0.90, 0.82, respectively), left ventral striatum (*d* = 0.88, 0.67), bilateral medial orbitofrontal cortex (*d* = 0.86, 0.71; 0.90, 0.73), and left inferior temporal gyrus (*d* = 0.83, 0.66), and decreased volumes in left premotor/presupplementary motor areas (*d* = − 0.83, − 0.71). Interestingly, abnormalities in the thalamus and medial orbitofrontal cortex were present in medicated patients whereas entirely absent in premotor and ventral striatum. It suggests that pharmacotherapy elicited divergent responses in orbitofronto-striato-thalamic and premotor circuits, which warrants the design of longitudinal studies investigating the potential of these neuromarkers in stratified treatments of OCD.

## Highlights

• Drug-naïve OCD patients have increased limbic and decreased associative regional gray matter volumes compared to controls.
• Pathological alterations of the structural basis of in the limbic and associative networks diverge in patients with OCD.
• First-line pharmacological interventions may selectively modulate different domains of neural circuits in OCD.

Pharmacotherapies including the use of selective serotonin reuptake inhibitors are considered as first-line interventions for OCD. Despite their long-history utility, nearly half of patients with OCD fail to respond adequately. Moreover, the therapeutic neural circuit mechanisms remain elusive due to insufficient statistical power and poor reproducibility. Here, we integrated structural MRI technique with rigorous Monte Carlo cross-validation analysis in a large grouped cohort to examine pharmacologically modulated volumetric alterations in OCD-specific pathological circuits. This cross-validated dissection of circuit-level therapeutic effects provides new insights into advancing stratified medicine in patients with characteristic neuroanatomical deficits, thereby improving treatment efficacy in OCD.

## 1 Introduction

Although rapid advances in structural neuroimaging studies using voxel-based morphometry (VBM) have enabled systematic assessment of the structural substrates underlying obsessive-compulsive related disorders (Ashburner and Friston, 2000, Lerch et al., 2017), our understanding of these pathological alterations remains shaded by conflicting and inconclusive prior findings. These apparent discrepancies may partially be due to insufficient power, clinical heterogeneity, mixed statistical analytic methods and medication confounds (Abi-Dargham and Horga, 2016, Blackford, 2017, Button et al., 2013, Poldrack et al., 2017, van den Heuvel et al., 2009). Among those confounding factors, pharmacological intervention has thus far received insufficient attention, as yields extremely limited (yet still diverging) evidence regarding the modulatory activity of therapeutics such as selective serotonin reuptake inhibitors (SSRIs) in OCD (Pine and Freedman, 2017, Skapinakis et al., 2016). Early studies selectively analyzed abnormally increased volumes of the thalamus and amygdala in drug-naïve pediatric patients, both of which were normalized by pharmacotherapy (Gilbert et al., 2000, Szeszko et al., 2004). Two whole-brain VBM studies on over a dozen patients found volume reductions in the left putamen (Hoexter et al., 2012) and parietal lobes (Lazaro et al., 2009) that became comparable to controls after medication. To date, the underlying circuit-level therapeutic mechanisms of pharmacotherapy remain essentially unclear while as many as half of patients diagnosed with OCD fail to respond adequately to serotonergic-based drugs (Bloch et al., 2006, Bloch et al., 2010, Hirschtritt et al., 2017). Understanding the brain-modulatory effects of medication treatment may help to match the pathological deficits in brain morphology of patients with therapeutic network fingerprints of specific drugs, thereby leading to improved treatment efficacy.

It is known that evidence generated from small sample sizes is especially prone to error: both false negatives due to inadequate power and false positives due to biased samples (Button et al., 2013). Meta- and mega-analysis of VBM studies combining data from independent studies can substantially improve statistical power, and have demonstrated that brain abnormalities in OCD not only manifest in the orbitofronto-striato-thalamic circuits, but also extend to other regions such as the dorsolateral prefrontal cortex, premotor and parietal areas (Carlisi et al., 2016, de Wit et al., 2014, Eng et al., 2015, Norman et al., 2016, Radua et al., 2010, Rotge et al., 2009, Rotge et al., 2010). Nevertheless, even in these large-sample reports, considerable inconsistency with regard to the presence and significance of anatomical abnormalities still exists. For instance, gray matter volume (GMV) of the ventral striatum (VS) has been described as increased (Carlisi et al., 2016, Norman et al., 2016), and unchanged (Eng et al., 2015, Radua et al., 2010, Rotge et al., 2010) in OCD compared to healthy controls. Volume sizes of the dorsolateral prefrontal cortex (dlPFC) and premotor areas were found to be decreased (Carlisi et al., 2016, Norman et al., 2016, Rotge et al., 2010) or normal (Eng et al., 2015, Radua et al., 2010). The validity of meta-analyses heavily relies on the methodological quality of the included studies, the eligibility criteria used for the meta-analysis, and various reporting biases (Finckh and Tramer, 2008, Walkup, 2017). Effect sizes, estimates of population parameters that are independent of sample size and other design decisions, provide a tool for determining whether a finding is not only statistically significant, but also whether a detected difference is substantive (Blackford, 2017). To test the internal validity within a sample and the external validity across multiple samples, cross-validation methods are usually required to provide final parameter estimates that are less biased and more likely to be replicated in independent future studies.

In this study, we sought to robustly evaluate the regional volumetric brain abnormalities of OCD and to determine the modulation of these pathological alterations by pharmacological treatment. We employed a cross-group design with a total of 328 participants that included three subgroups of patients (drug-naïve, medicated and medication-free) and a group of healthy comparison (HC) subjects. We first identified pathological differences between drug-naive patients and HCs at the whole-brain scale. We then focused on OCD-specific pathological regions to investigate disorder-related pharmacological effects in the medicated group. To demonstrate a robust association of brain volumetric changes with pharmacotherapy, we tested whether the observed structural responses to medication existed in the third sample of medication-free adult patients who had discontinued medication (at least 4 weeks prior). Considering clinical heterogeneity related to variation in disease profile and treatment trajectory, a Monte Carlo cross-validation (MCCV) procedure was employed at each group comparison and the effect sizes were reported correspondingly. Potential modulating effects of demographic, clinical and statistical characteristics on brain volume were regressed out of the present results. With fully cross-validated analysis, we thus expected to identify reliable and reproducible structural substrates of OCD pathophysiology and their network signatures in response to pharmacological therapies.

## 2 Materials and Methods

### 2.1 Participants

Between April 2, 2013 and April 13, 2016, patients were recruited through local inpatient and outpatient departments at the OCD Clinics at Ruijin Hospital and Shanghai Mental Health Center. All participants provided written informed consent for study participation after receiving a complete description of the protocols, which were approved by the Institutional Review Boards at Ruijin Hospital and Shanghai Mental Health Center, Shanghai Jiao Tong University and by the Biomedical Research Ethics Committee, Shanghai Institutes for Biological Sciences, and Chinese Academy of Sciences. All patients had received a primary diagnosis of OCD based on clinical evaluation with the Chinese translation of the Structured Clinical Interview for DSM-IV-TR, and were administered the Yale-Brown Obsessive Compulsive Scale (Y-BOCS) to assess OCD symptom severity (Goodman et al., 1989). 233 OCD patients (Five patients who were under the age of 18 were excluded for subsequent analysis) were categorized into 3 subgroups: drug-naïve OCD (*n* = 95), medicated OCD (*n* = 65), and medication-free OCD (*n* = 73). Approximately 97% of OCD patients in the medicated subgroup had received clinical treatment with SSRIs (see Table S1). Patients who had discontinued medication for at least four weeks were considered medication-free. 95 healthy subjects were recruited through local advertisements. Exclusion criteria included age below 18 or over 65 years, any neurological disorders, psychosurgery, current or past substance abuse or dependence, pregnancy or any substantial physical illness such as brain tumor, brain injury, stroke, or epilepsy.

Demographic and clinical data were analyzed for group differences using one-way analysis of variance (ANOVA) and post hoc least significant difference tests (SPSS, version 22). Gender and handedness ratios for each group were analyzed with chi-square and Fisher's exact tests, respectively (Table 1).

**Table 1:** Demographic and clinical characteristics of patients with OCD and healthy comparison subjects.

Characteristics	Drug-naïve (*n* = 95)	Medicated (*n* = 65)	Medication-free (*n* = 73)	Healthy controls (*n* = 95)	Analysis	
Demographic					F (df = 3324)^a^	*p*
Age, years	29.97 (8.28)	30.63 (8.68)	32.08 (8.94)	30.42 (10.81)	0.76	0.52
Education, years	14.66 (2.90)	12.68 (3.37)	13.71 (3.22)	15.14 (3.65)	8.15	< 0.001
Sex, female	43 (45)	29 (45)	32 (44)	46 (48)		0.94^b^
Handedness, right	95 (100)	64 (98)	72 (99)	93 (98)		0.66^c^

Clinical					F (df = 2230)^a^	*p*
Age of OCD onset, years	22.58 (8.79)	20.33 (8.34)	23.34 (10.26)	–	2.00	0.14
Yale-Brown Obsessive Compulsive Scale						
Total score	25.64 (5.25)	27.46 (7.50)	26.53 (5.11)	–	1.84	0.16
Obsessions	13.31 (3.20)	15.91 (3.86)	13.99 (3.03)	–	11.90	< 0.001
Compulsions	12.33 (3.77)	11.55 (6.69)	12.55 (3.64)	–	0.83	0.44

Data are mean (SD), *n* (%).

a One-way ANOVA (df = 3324 for demographic variables test; df = 2230 for clinical variables test, *p* < 0.05, two-tailed).

b Chi-square test.

c Fisher's exact test.

### 2.2 Image Acquisition and Processing

All subjects were scanned using a Siemens Tim Trio 3 T scanner or Siemens Verio scanner (Erlangen, Germany). High-resolution T1-weighted anatomical images were acquired using a 3D magnetization-prepared rapid gradient-echo sequence (repetition time = 2300 ms, TE = 3 ms, TI = 1000 ms, flip angle = 9°, voxel size = 1.0 × 1.0 × 1.0 mm^3^). The data were processed in SPM8 (http://www.fil.ion.ucl.ac.uk/spm) and VBM8 toolbox (http://dbm.neuro.uni-jena.de/vbm/). All MRI images were visually inspected before data processing to exclude scans exhibiting gross brain pathology, artifacts, or reduced image quality hampering image segmentation. An automated quality check using covariance analysis on the sample homogeneity of segmented GM images was performed in parallel to visual inspection, and did not lead to participant exclusion. T1-weighted images were (a) corrected for field inhomogeneities; (b) registered using a DARTEL (diffeomorphic anatomical registration through exponentiated lie algebra) template (Ashburner, 2007); (c) stripped of non-brain tissue; (d) tissue-segmented into gray matter, white matter and cerebrospinal fluid; (e) modulated for different tissue segments to preserve the regional volumetric information of a particular tissue within a voxel. This was done by multiplying the intensity value of each voxel in the segmented images by the Jacobian determinants (non-linear components only) that were derived from the spatial registration process. To increase the signal-to-noise ratio, images were smoothed with an 8-mm isotropic Gaussian kernel. A gray matter mask was applied before statistical comparison, which was derived from the Desiken-Killinay atlas (Desikan et al., 2006) and contained 34 cortical regions per hemisphere, 14 subcortical regions and the cerebellum (see Table S2).

### 2.3 Statistical Analysis and Cross Validation

Group effect on regional GMV was investigated by feeding the processed MRI images of drug-naïve OCD and HCs into general linear models that removed the confounding effects of age, sex, education level and total GMV. Clusters of voxels were considered statistically significant if the results of group comparison passed an uncorrected p threshold of 0.005 and withstood Monte Carlo cross-validation (MCCV). The MCCV procedure (repeated random sub-sampling validation) was performed by randomly selecting one fraction of the original data sample (60% of each group here) at each time and then conducting a group comparison as described above. This process was repeated 10,000 times; results of voxels with *p* < 0.005 that had occurrences exceeding 95% and a cluster size > 25 were considered significant. As a methodological validation, we also report the statistical results of group differences in regional volumes using family-wise error (FWE) correction for multiple comparisons. During each group comparison of regional volume, the same MCCV procedure was applied and the effects of factors involving age, gender, education level, and total GMV were regressed out as covariates. In addition to the common *p* value measure of statistical significance, we report Cohen's *d* value as a measure of effect size. Effect sizes were divided into three levels: small, medium, and large, each corresponding to a Cohen's *d* value greater than or equal to 0.2, 0.5, and 0.8. We calculated Cohen's *d* using the means and standard deviations of two groups for each comparison across 10,000 MCCV. We considered values to be pragmatically significant when they demonstrated both statistical significance (occurrences of *p* < 0.05 exceeding 95%) and substantial significance (a sizable effect size indicated by occurrences of |* d* | ≥ 0.5 with 95% confidence interval not including 0 in over 95% of validation rounds). For regions with significant group differences and sizable effect sizes between drug-naïve OCD and controls, we compared mean regional GMVs between the medicated patient group and controls, and then re-examined these differences in the medication-free group using a two-sample *t*-test. Finally, partial correlation analyses with age, sex, education level and total GMV as covariates were used to examine the relationship between volumetric changes and OCD symptom severity.

## 3 Results

### 3.1 Demographic and Clinical Characteristics

The characteristics and clinical assessments for patients with OCD and HCs are summarized in Table 1. There was a significant group effect of OCD obsession symptom severity (ANOVA, *F*_2230_ = 11.90, *p* < 0.001). Post hoc tests confirmed that the medicated OCD group had a significantly larger Y-BOCS obsession score than the drug-naïve OCD group (*p* < 0.001) and medication-free OCD group (*p* = 0.001). Patients and comparison subjects did not differ significantly in distributions of age, sex and handedness (all *p* > 0.05). The groups did not differ significantly in total GMV (drug-naïve OCD: mean = 675 cm^3^, SD = 73; medicated OCD: mean = 657 cm^3^, SD = 69; medication-free OCD: mean = 665 cm^3^, SD = 62; HC: mean = 656 cm^3^, SD = 58) (ANOVA, F_3324_ = 1.66, *p* = 0.18). One-way ANOVA showed a significant group effect of educational level (ANOVA, *F*_3324_ = 8.15, *p* < 0.001). Post hoc tests confirmed that the control group had a higher educational level than the medicated group (*p* < 0.001) and the medication-free group (*p* < 0.001). The drug-naïve group had a higher educational level than the medicated group (*p* < 0.001). Potential modulating effects of demographic and clinical characteristics on brain volume were regressed out of the present results, although we did not strictly control demographic distributions of each group during 10,000 MCCV.

### 3.2 Association of Volumetric Changes With Drug-Naïve OCD

**Fig. 1:**
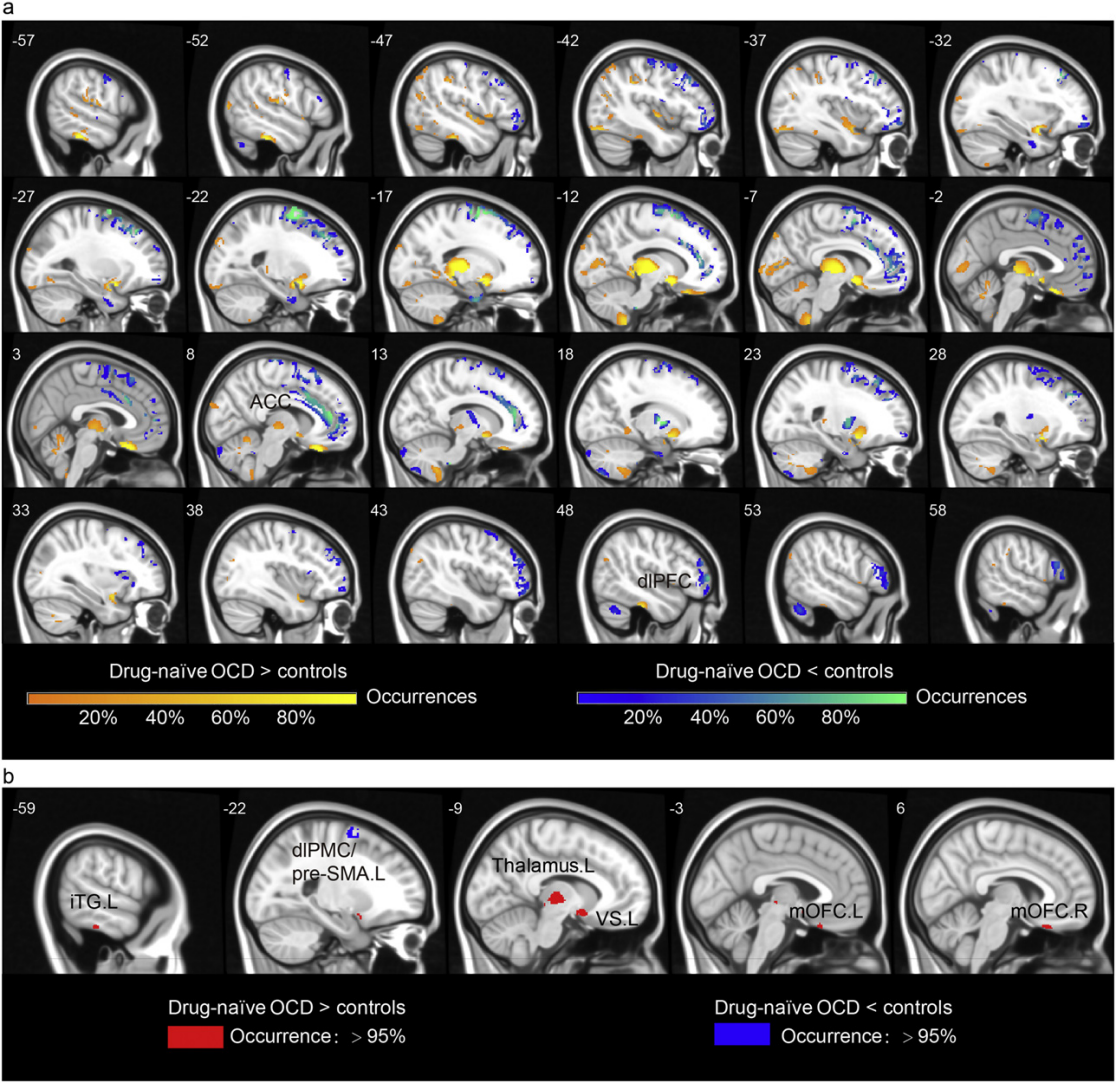
Regional GMV differences between drug-naïve patients with OCD and healthy controls. (a) Shows how sample heterogeneity dynamically impacts the results of group comparison using Monte Carlo Cross Validation (MCCV). The MCCV was repeated 10,000 times by randomly selecting one fraction of the original data sample (60% of each group). Color bars indicate results that had occurrences of significant voxels (*p* < 0.005, uncorrected) from 10% to 100% throughout 10,000 MCCV rounds. (b) Results with occurrences exceeding 95% were considered statistically significant. Compared to controls, drug-naïve patients with OCD exhibited increased (red region) regional GMVs in the left thalamus, left VS, bilateral mOFC, left iTG and decreased (blue region) GMVs in the left dlPMC/pre-SMA (see Table S3 for detailed information). Numbers denote z coordinates in standard MNI space. GMV, gray matter volume; VS, ventral striatum; mOFC, medial orbitofrontal cortex; iTG, inferior temporal gyrus; dlPMC/pre-SMA, dorsolateral premotor cortex/presupplementary motor area; R, right; L, left. (For interpretation of the references to color in this figure legend, the reader is referred to the web version of this article.)

Fig. 1a shows how regions with significant group difference between drug-naïve OCD and HC varied by data composition, illustrated as the number of occurrences in 10,000 repetitions (color scale: 10%–100%). When occurrence probability is thresholded to include regions that achieved significance in over 95% of MCCV rounds, Fig. 1b shows significantly increased regional GMVs in the left thalamus, left VS including nucleus accumbens and ventral putamen, bilateral medial orbitofrontal cortex (mOFC) and left inferior temporal gyrus (iTG), but significantly reduced in the left dorsolateral premotor/presupplementary motor area (dlPMC/preSMA) (see Table S3 for details of each region). Note that decreased GMV of the bilateral anterior cingulated cortex (ACC) and dlPFC with relatively high occurrences of significant voxels during MCCV (Fig. 1a) failed to withstand a stringent threshold (Fig. 1b), suggesting that pathological differences observed in these two regions are highly susceptible to variation in data constituents. We further used the FWE correction for multiple comparisons to validate these regional morphometric alterations (see Table S4). There was a significant correlation between the mean GMV of left ventral striatum (VS) and the Y-BOCS compulsion scores (*p* = 0.0035, partial correlation coefficient ρ = − 0.35) in drug-naïve OCD group.

### 3.3 Association of Volumetric Changes With Pharmacotherapy

Among the six regions identified above, only the left mOFC exhibited a significant difference in medicated patients as compared to the control group, with 95.80% occurrence at *p* < 0.05 (Table 2). Although statistically significant differences in the regional GMV of the left thalamus, left iTG and right mOFC occurred at probabilities over 50%, their effect sizes were essentially small throughout 10,000 MCCV. The chances of observing significant differences in the other regions, including the left VS, and left dlPMC/pre-SMA were rather low (Table 2). By contrast, in the medication-free OCD group, we found significant volume increases in the left thalamus, left VS, bilateral mOFC, left iTG, and volume reduction of the left dlPMC/pre-SMA (occurrences at *p* < 0.05 exceeding 95%) (Table 2). These findings were consistent with observations in drug-naïve OCD patients, and contrary to observations in medicated patients.

**Table 2:** Group-comparison results of statistical significance and effect size across 10,000 MCCV process.

Regions	Drug-naïve vs. controls			Medicated vs. controls			Medication-free vs. controls		
	Mean *d*	*p* < 0.05	|* d* | ≥ 0.5	Mean *d*	*p* < 0.05	|* d* | ≥ 0.5	Mean *d*	*p* < 0.05	|* d* | ≥ 0.5
Thalamus.L	0.90 (0·12)	**100%**	**99.97%**	− 0.47 (0.14)	77.07%	40.13%	0.82 (0.12)	**100%**	**99.51%**
VS.L	0.88 (0·12)	**100%**	**99.98%**	− 0.26 (0.14)	14.92%	4.14%	0.67 (0.13)	**99.30%**	91.74%
mOFC.R	0.90 (0·13)	**99.99%**	**99.95%**	− 0.55 (0.12)	79.21%	65.22%	0.73 (0.14)	**98.88%**	**95.64%**
mOFC.L	0.86(0.12)	**99.98%**	**99.88%**	− 0.64(0.14)	**95.80%**	84.58%	0.71(0.13)	**97.46%**	**94.60%**
iTG.L	0.83 (0·13)	**100%**	**99.75%**	− 0.41 (0.13)	55.80%	26.12%	0.66 (0.13)	**98.84%**	89.04%
dlPMC.L	− 0.83 (0·13)	**100%**	**99.81%**	− 0.04 (0.14)	1.36%	0.00%	− 0.71 (0.13)	**99.69%**	**95.06%**

Data are mean (SD) or %. GMV, gray matter volume; MCCV, Monte Carlo cross-validation; VS, ventral striatum; mOFC, medial orbitofrontal cortex; iTG, inferior temporal gyrus; dlPMC, dorsolateral premotor area; R, right; L, left.

Bold indicates more than 95% occurrences in MCCV process.

**Fig. 2:**
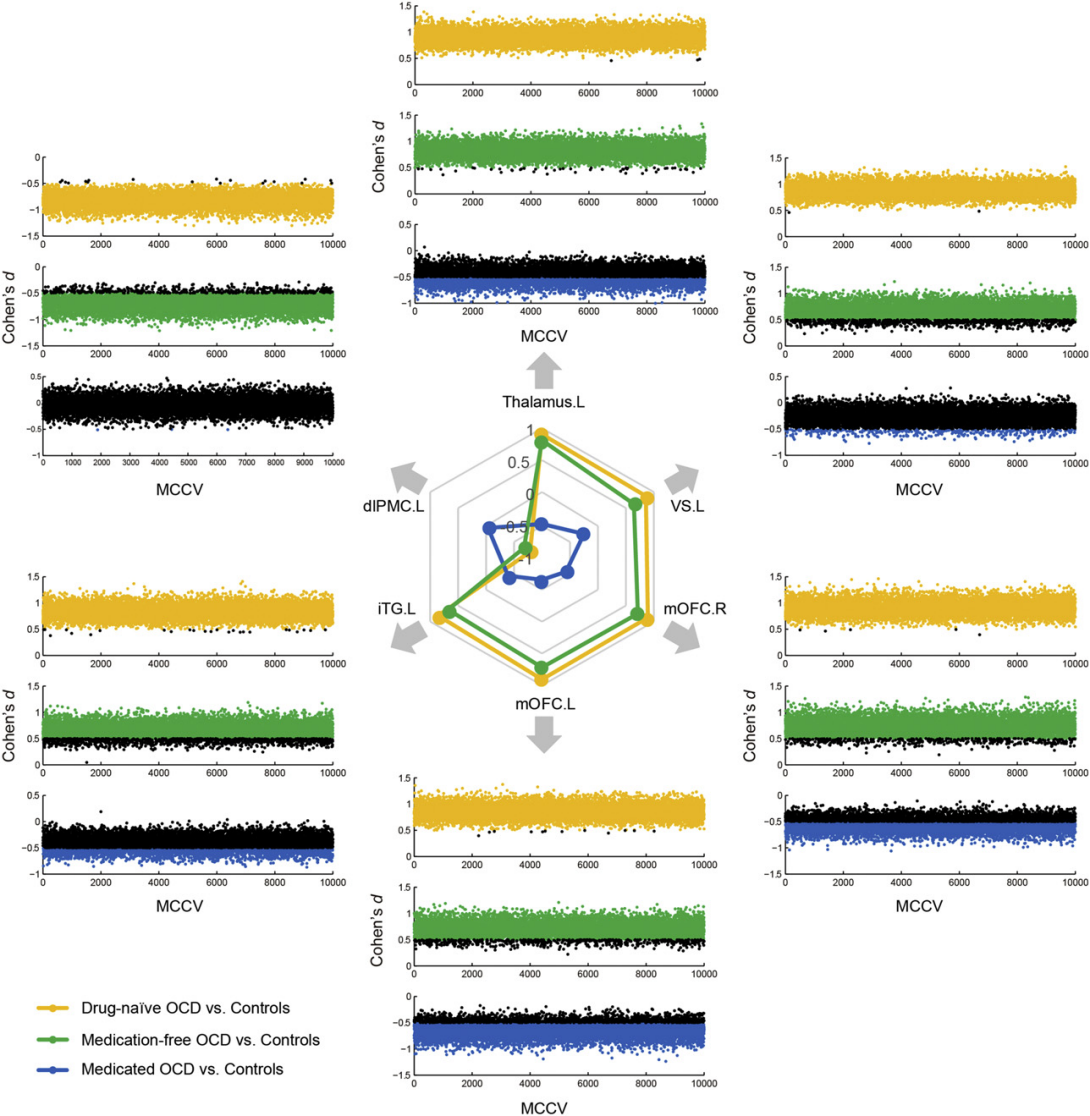
Association of brain volumetric changes with pharmacotherapy in OCD. In the spider plot (shown in the center), mean values of Cohen's *d* across 10,000 MCCV of three OCD subgroups (drug-naïve, medicated, and medication-free) as compared to controls are plotted as scales, demonstrating that OCD-specific GMV abnormalities (yellow line) are selectively modulated by medication (blue line) but re-emerge in the medication-free condition (green line). Scatter plots of six key regions show how their effect sizes varied across 10,000 MCCV for three subgroups. Effect sizes of both drug- naïve and mediation-free subgroups appear to be fairly consistent across 10,000 MCCV rounds (|* d* | ≥ 0.5 exceeding 95%), in contrast to the medicated subgroup (see main text and Table 2 for more information). In all scatter plots, black dots indicate |* d* | < 0.5. VS, ventral striatum; mOFC, medial orbitofrontal cortex; iTG, inferior temporal gyrus; dlPMC, dorsolateral premotor cortex; R, right; L, left. (For interpretation of the references to color in this figure legend, the reader is referred to the web version of this article.)

We continued to cross-validate the effect sizes of these statistically significant findings. In Fig. 2, a spider plot is generated on the basis of the mean Cohen's *d* values of all six regions (shown in Fig. 1b) with significant GMV changes in the drug-naïve, medicated and medication-free groups, as confirms the volumetric changes induced by pharmacotherapy. To demonstrate whether the measure of effect size is robust with regard to data composition, we plotted all Cohen's *d* values throughout 10,000 MCCV for all six regions in three group comparisons (Fig. 2). Effect sizes for these regions are markedly consistent during cross-validation in the drug-naïve and medication-free groups, but not in the medicated group (black dots denote the effect sizes smaller than 0.5, Fig. 2). There was a significant relationship between the mean GMV of left iTG and Y-BOCS obsession scores (*p* = 0.0098, partial correlation coefficient ρ = 0.30) in the medication-free OCD group.

## 4 Discussion

Despite the abundance of research investigating structural abnormalities and their treatment-related changes in OCD, previous reports rarely make reliable distinctions between volumetric characteristics associated with the disease process and treatment-related structural responses (Abi-Dargham and Horga, 2016, Bloch et al., 2006, Skapinakis et al., 2016). This imposes substantial restrictions on the clinical relevance and future applicability of neuroimaging findings. The structural profile of brain abnormalities associated with OCD without medication confounds is critically important for stratified medicine in future clinical practice, in which imaging-based neuromarkers predicting therapeutic response are matched to the pathological circuits identified in a subpopulation of patients (Abi-Dargham and Horga, 2016). In both drug-naïve and medication-free cohorts, we identified internally- and externally-validated morphologic alterations mainly in the limbic network including the mOFC and VS, and the associative network including premotor/pre-SMA areas.

We found marked increases in GMVs of the VS and mOFC. As a key region in the orbitofronto-striato-thalamic pathway (Menzies et al., 2008, Milad and Rauch, 2012, Pauls et al., 2014), VS (mainly the nucleus accumbens in this study) demonstrated significant enlargement as in previous studies (Norman et al., 2016, Pujol et al., 2004), while existing reports show either increased (Szeszko et al., 2008) or decreased volumes in OFC (Norman et al., 2016, Rotge et al., 2009). Functional studies have indicated that hyperactivation in the mOFC and caudate may be related to goal-directed dysfunction in OCD (Gillan et al., 2014). Meanwhile, the thalamus as a central link in CSTC circuitry demonstrated abnormally increased volume, a finding consistently associated with OCD pathology here and in other studies (Boedhoe et al., 2016, Eng et al., 2015, Rotge et al., 2009). In contrast, decreased GMVs of the left dlPMC/pre-SMA are observed, as in prior meta-analyses of OCD studies (Norman et al., 2016, Rotge et al., 2009). Premotor areas are critical for response inhibition, both in suppressing an unwanted action and facilitating a desired one (Duque et al., 2012). Recently de Wit and colleagues found left dlPMC/pre-SMA hyperactivity in OCD patients and their unaffected siblings during response inhibition (de Wit et al., 2012). Interestingly, OCD patients not only exhibit increased attention to the actual outcomes of such actions, but also show heightened intra-individual correlation between error-related negativity and activity in pre-SMA that is consistent with increased worry about future negative outcomes (Grutzmann et al., 2016).

In order to glean clinically meaningful volume alterations caused by pharmacotherapy, we integrated effect size analysis with rigorous cross-validation procedures, revealing strikingly differential effects of pharmacological modulation on OCD-specific abnormalities. Firstly, trivial differences remained between the medicated and control groups in premotor and ventral striatum areas, indicating that the most prominent therapeutic effects of serotonergic-based drugs occur in these regions. Secondly, the modulatory effect on the thalamus is relatively ambiguous here, as significant medication-related differences occurred in over 77% of sampling rounds (albeit with small effect sizes; Table 2) despite earlier longitudinal accounts revealing reversible volumetric changes induced by SSRI treatment (Gilbert et al., 2000, Hoexter et al., 2012, Szeszko et al., 2004). Lastly, we unveiled that the orbitofrontal cortex of OCD patients, particularly the medial part of the left hemisphere, exhibits pronounced resistance to pharmacological modulations. Recent evidence based on animal models has shown that hyperstimulation of glutamatergic OFC-ventromedial striatum projections leads to compulsive-like grooming behavior that is reversible with chronic fluoxetine, further substantiating its potential as a therapeutic target for OCD (Ahmari et al., 2013). Intriguingly, our analysis implies divergent pharmacological modulation of the OFC and ventral striatum, which requires further experimental investigation to dissect their distinct predictive responses in the orbitofronto-striato-thalamic loop.

It is worth mentioning that prioritized alterations in brain regions and circuits imposed by various medications are a core characteristic of clinical biomarkers (Abi-Dargham and Horga, 2016). The key medication targets identified here overlap partially with regions preferentially targeted by an emerging treatment candidate – ketamine, which mainly acts on the OFC, subgenual and posterior cingulate cortices, and nucleus accumbens, as demonstrated in our recent preclinical work (Lv et al., 2016). Collectively, these findings raise promising potential for the development of specific loci or circuit-targeted therapeutics that are potentially more effective in particular subpopulations of patients involving particular phenotypic/dimensional deficits.

Growing concerns about the robustness and reproducibility of neuroimaging results have recently garnered unanimous attention (Abi-Dargham and Horga, 2016, Blackford, 2017, Lerch et al., 2017, Poldrack et al., 2017). The quest for pragmatically usable biomarkers calls for the rigorous assessment of the strength of an effect beyond its statistical significance, including its reliability within a sample, as well as generalizability across samples (Abi-Dargham and Horga, 2016, Poldrack et al., 2017). To confront these concerns, we performed internal validation in the largest sample of drug-naïve OCD (until now), using a repeated random sub-sampling (i.e., MCCV) strategy and reported effect sizes. We demonstrated that both statistically significant results and effect sizes are substantially vulnerable to the heterogeneity of the patient population (Figs. 1a and 2). For example, while decreased volumetric sizes in the ACC and dlPFC have been reported in prior studies (de Wit et al., 2014, Norman et al., 2016, Radua et al., 2010), their occurrence throughout 10,000 MCCV rounds was slightly lower than the threshold predefined here (95%). We hypothesize that the contribution of ACC and dlPFC to the pathophysiology of OCD may delineate a symptomatically and possibly etiologically specific subgroup of the patient population (Gillan et al., 2014, Milad and Rauch, 2012, van den Heuvel et al., 2009). We also found the effect sizes of the six identified disease-specific areas to be reproducible in both drug-naïve and medication-free groups relative to controls and consistent throughout 10,000 MCCV. In the medicated group, however, these key regions exhibited remarkably varied responses to pharmacological treatments, particularly in the premotor and VS areas versus mOFC and thalamus. The present cross-validated observations may also offer an explanation to reconcile prior discrepant empirical findings. Nevertheless, the results reported herein must be interpreted with caution due to several practical limitations. This is only a cross-sectional study, though it was conducted across multiple samples. We were unable to directly evaluate the symptom improvements mediated by medications, such that dose-dependent structural differences modulated by drug treatment to patients with OCD were not determined in the current settings (Bloch et al., 2010). These issues certainly merit future investigation.

In summary, we present the first cross-validated study to demonstrate a reliable, reproducible association of volumetric alterations in a total of 233 OCD patients with and without pharmacological intervention. Our results reveal six major regions of both increased and decreased volume sizes in multiple OCD groups as compared to controls, and that first line drug medication (mainly serotonergic-based therapeutics) may biasedly modulate premotor and ventral striatum areas efficiently, with less effectiveness in thalamus and medial orbitofrontal cortical areas. The present findings provide novel insights into how pharmacotherapeutic action shapes neuroanatomical circuits in OCD, and may also account for previous discrepant findings.

## Funding Sources

This work was supported by the Hundred Talent Program of the Chinese Academy of Sciences (Technology), Strategic Priority Research Program (B) of the Chinese Academy of Sciences (XDB02050000) and National Natural Science Foundation of China Grant (81571300, 81527901 to Z.W.; 81271518 and 81471387 to B.M.S.).

## Conflicts of Interests

All authors declare no conflict of interest.

## Author Contributions

Z.W. (Zheng Wang) and Z.P.X were responsible for the concept and the design of the study. Q.M.L., Z.W. (Zhen Wang), C.C.Z., Q.F., and Q.Z. acquired the data. Q.M.L., Z.W. (Zhen Wang), C.C.Z., Q.F., Q.Z., and K.Z. analyzed all or parts of the data. Q.M.L., Z.W. (Zhen Wang), C.C.Z., B.M.S., Z.P.X, and Z.W. (Zheng Wang) drafted all or parts of the article, with input from other authors. Z.W. (Zheng Wang) and B.M.S. obtained the funding and supervised the study.

## Appendix A Supplementary Data

Supplementary tables Image 1
