# Supplementary material for: Divergent Structural Responses to Pharmacological Interventions in Orbitofronto-Striato-Thalamic and Premotor Circuits in Obsessive-Compulsive Disorder
Source: eBioMedicine. 2017 Jul 26;22:242–8. doi: 10.1016/j.ebiom.2017.07.021 (PMC5552245; doi:10.1016/j.ebiom.2017.07.021)
Supplement: Supplementary file 1 — Supplementary tables [file mmc1.docx]

**Supplementary tables**

| **Table S1: Medication summary for the medicated OCD group (n=65)** | | |
| --- | --- | --- |
| Mechanism | Drug | n (%) |
| SSRI | Paroxetine | 27 (42) |
|  | Fluvoxamine | 22 (34) |
|  | Fluoxetine | 21 (32) |
|  | Sertraline | 21 (32) |
|  | Citalopram | 6 (9) |
|  | Escitalopram | 3 (5) |
| TCA | Clomipramine | 16 (25) |
| SNRI | Venlafaxine | 4 (6) |
|  | Duloxetine | 3 (5) |
| Benzodiazepine | Clonazepam | 4 (6) |
| Atypical antipsychotic | Olanzapine | 8 (12) |
|  | Quetiapine | 7 (11) |
|  | Clozapine | 4 (6) |
|  | Ziprasidone | 3 (5) |
|  | Aripiprazole | 3 (5) |
|  | Sulpiride | 2 (3) |
|  | Risperidone | 2 (3) |
| Data are n (%). Medication history was unknown for one subject. Some patients were treated with more than one drug upon clinical need (41%). Drugs including sodium valproate, flupentixaol/melitracen, mirtazapine, amitriptyline, trazodone were taken by no more than one patient. Sixty-four out of sixty-six patients had received SSRI treatment (97%); SSRI, selective serotonin reuptake inhibitor; SNRI, serotonin–norepinephrine reuptake inhibitor; TCA, tricyclic antidepressant. | | |

| **Table S2: Regions used for gray matter mask** | | |
| --- | --- | --- |
| Labels | Regions | Hemisphere |
| 1 | Bank of the superior temporal sulcus | L/R |
| 2 | Caudal anterior cingulate cortex | L/R |
| 3 | Caudal middle frontal cortex | L/R |
| 4 | Cuneus | L/R |
| 5 | Entorhinal cortex | L/R |
| 6 | fusiform | L/R |
| 7 | Inferior parietal cortex | L/R |
| 8 | Inferior temporal cortex | L/R |
| 9 | Isthmus of the cingulate cortex | L/R |
| 10 | Lateral occipital cortex | L/R |
| 11 | Lateral orbitofrontal cortex | L/R |
| 12 | Lingual gyrus | L/R |
| 13 | Medial orbitofrontal cortex | L/R |
| 14 | Middle temporal cortex | L/R |
| 15 | Parahippocampal cortex | L/R |
| 16 | Paracentral gyrus | L/R |
| 17 | Pars opercularis | L/R |
| 18 | Pars orbitalis | L/R |
| 19 | Pars triangularis | L/R |
| 20 | Pericalcarine cortex | L/R |
| 21 | Postcentral gyrus | L/R |
| 22 | Posterior cingulate cortex | L/R |
| 23 | Precentral gyrus | L/R |
| 24 | Precuneus | L/R |
| 25 | Rostral anterior cingulate cortex | L/R |
| 26 | Rostral middle frontal cortex | L/R |
| 27 | Superior frontal cortex | L/R |
| 28 | Superior parietal cortex | L/R |
| 29 | Superior temporal cortex | L/R |
| 30 | Supramarginal gyrus | L/R |
| 31 | Frontal pole | L/R |
| 32 | Temporal pole | L/R |
| 33 | Transverse temporal cortex | L/R |
| 34 | Insula | L/R |
| 35 | Thalamus | L/R |
| 36 | Caudate | L/R |
| 37 | Putamen | L/R |
| 38 | Pallidum | L/R |
| 39 | Hippocampus | L/R |
| 40 | Amygdala | L/R |
| 41 | Accumbens area | L/R |
| 42 | Cerebellum | - |
| Regions used for the gray matter mask were derived from the Desiken-Killinay atlas, and included 34 cortical ROIs and 14 subcortical ROIs per hemisphere, and cerebellum. | | |

| **Table S3: Regional GMV differences between drug-naïve patients with OCD (n=95) and healthy controls (n=95) controlled by MCCV** | | | | | | |
| --- | --- | --- | --- | --- | --- | --- |
|  |  |  | **MNI Coordinates** | | |  |
| **Anatomical Region** | **Side** | **BA** | **x** | **y** | **z** | **Cluster Size** |
| *Drug-Naïve OCD > controls* |  |  |  |  |  |  |
| Thalamus | L | - | -9 | -24 | -3 | 683 |
| VS (NAcc and putamen) | L | - | -12 | 6 | -12 | 237 |
| mOFC | R | 11 | 8 | 27 | -27 | 84 |
|  | L | 11 | -3 | 21 | -26 | 27 |
| iTG | L | 20 | -59 | -35 | -27 | 52 |
| *Drug-Naïve OCD < controls* |  |  |  |  |  |  |
| dlPMC/pre-SMA | L | 6 | -21 | -2 | 65 | 152 |
| Monte Carlo Cross-Validation (MCCV) procedure. Results of the MCCV had occurrences of significant voxels exceeding 95% and a cluster size greater than 25 in two sample *t*-tests. BA, Brodmann’s area; MNI, Montreal Neurological Institute; VS, ventral striatum; NAcc, nucleus accumbens; mOFC, medial orbitofrontal cortex; iTG, inferior temporal gyrus; dlPMC, dorsolateral premotor area; pre-SMA, presupplementary motor area; R, right; L, left. | | | | | | |

| **Table S4: Regional GMV differences between drug-naïve patients with OCD (n=95) and healthy comparison subjects (n=95)** **controlled by FWE** | | | | | | | | |
| --- | --- | --- | --- | --- | --- | --- | --- | --- |
|  |  |  | **MNI Coordinates** | | |  |  |  |
| **Anatomical Region** | **Side** | **BA** | **x** | **y** | **z** | **Cluster Size** | **t_(df=184)_** | **p_(FWE)_** |
| *Drug-Naïve OCD > controls* |  |  |  |  |  |  |  |  |
| Thalamus | L | - | -14 | -15 | -2 | 793 | 7.76 | 0.000 |
|  |  |  | -11 | -28 | -5 |  |  |  |
| VS | L | - | -12 | 6 | -12 | 293 | 5.88 | 0.000 |
|  |  |  | -27 | 8 | -18 |  |  |  |
| mOFC | R | 11 | 8 | 26 | -27 | 95 | 5.61 | 0.001 |
|  | L | 11 | -3 | -21 | -26 | 39 | 5.11 | 0.006 |
| iTG | L | 20 | -59 | -34 | -27 | 101 | 5.45 | 0.001 |
| *Drug-Naïve OCD < controls* |  |  |  |  |  |  |  |  |
| dlPMC/ pre-SMA | L | 6 | -21 | -3 | 63 | 146 | 5.39 | 0.002 |
| Results are corrected for age, gender, education level, total gray matter. Statistical threshold is set at p < 0.05, family-wise error (FWE) corrected. Total number of contiguous voxels in each region that surpassed the initial cutoff of 25. BA, Brodmann’s area; MNI, Montreal Neurological Institute; VS, ventral striatum; mOFC, medial orbitofrontal cortex; iTG, inferior temporal gyrus; dlPMC, dorsolateral premotor area; pre-SMA, presupplementary motor area; dACC, dorsal anterior cingulate cortex; R, right; L, left. | | | | | | | | |
